# Electronic cigarettes use and ‘dual use’ among the youth in 75 countries: estimates from Global Youth Tobacco Surveys (2014–2019)

**DOI:** 10.1038/s41598-022-25594-4

**Published:** 2022-12-05

**Authors:** Chandrashekhar T. Sreeramareddy, Kiran Acharya, Anusha Manoharan

**Affiliations:** 1 Department of Community Medicine, International Medical University, 126, Jalan Jalil Perkasa 19, Bukit Jalil, 57000 Federal Territory of Kuala Lumpur, Malaysia; 2 New ERA, Rudramati Marga, Kalopul, Kathmandu, 44621 Nepal; 3 Botanic Health Clinic, Ministry of Health, Selangor, Malaysia

**Keywords:** Epidemiology, Medical research

## Abstract

We report the country-level prevalence of awareness about electronic cigarette use, and ‘dual use’ and its association with age, sex, country income, and e-cigarette regulatory status. We analyzed the most recent Global Youth Tobacco Surveys done on nationally representative samples of school-going youth aged 13–15 years in 75 countries/territories. The weighted prevalence of ‘awareness’ (heard about e-cigarettes), ‘ever use’ (even tried a few puffs), ‘current use’ (during the last 30 days), and ‘dual use’ (e-cigarette use and cigarette smoking during the last 30 days) were estimated. Awareness was > 80% in 13 countries mostly from Europe Poland being the highest at 95.8% (95% CI 94.8- 96.6). In seven countries, 30–50% of the youth had ever used an e-cigarette, Italy was the highest at 55.1% (95%CI 51–3,58.9). In 30 countries, current e-cigarette use was > 10%, the highest of 35.1% (95%CI 32.4–38.0) in Guam. Awareness and use were highest in the European region (74.6% and 34.5%) and HIC (83.6% and 39.4%). Youth from HIC (compared to lMIC) and countries having restrictive e-cigarette regulations (compared to NRP) had 2.4 times (aOR 2.2.4, 95% CI 2.2, 2.7) and 1.8 times (aOR 1.8, 95% CI 1.6, 2.0) higher odds of being current e-cigarette users respectively. Youth in countries with the most restrictive e-cigarette regulations (compared to NRP) had 0.6 times lower odds of being current e-cigarette users (aOR 0.6, 95% CI 0.6, 0.7). Awareness and e-cigarette use varied by sex, country income level, and region. Continued global surveillance of youth e-cigarette use is needed for the formulation of e-cigarette regulatory policy. Awareness and use of e-cigarettes were higher among boys, in countries in Europe and America regions, and among those with higher income and restrictive policies, whereas it was lower in countries having the most restrictive policies. Higher awareness is strongly correlated with a trial and current use of e-cigarettes. E-cigarette marketing should be restricted, and continued surveillance of e-cigarette use is needed. Most restrictive policies such as the ban on e-cigarettes appear to reduce e-cigarette use among the youth.

## Introduction

Tobacco use is a major risk factor contributing to the preventable disease burden^1^. Global efforts guided by the Framework Convention for Tobacco Control^2^ resulted in the decline of tobacco use among adults^3,4^. Lifelong nicotine addiction if initiated at a younger age^5^ (this part seems incomplete) makes it imperative to prevent the uptake of smoking among the youth as a key strategy to end the tobacco epidemic^6^. Analysis from the Global Youth Tobacco Survey (GYTS) has shown that tobacco use excluding electronic cigarettes (e-cigarettes) among school-going youth aged 13–15 years still remains substantial^7^. E-cigarette use also known as ‘vaping’ is increasing among the youth particularly in high-income countries (HIC)^8^. Increased use of e-cigarettes by youth has raised concerns about the “trying out” of e-cigarettes and then taking up cigarette smoking, often referred to as the ‘gateway to smoking^9,10^. E-cigarette use has led to plateauing or decline in youth cigarette smoking in some countries^11,12^. Nevertheless, recent reports of increasing rates of ‘dual use’ (both cigarette smoking and e-cigarettes) suggests a higher level of dependence^13,14^. Data on medium- and long-term effects of e-cigarette use are lacking but evidence about potentially harmful health effects is growing^15,16^. This warrants public health actions to prevent the impending epidemic of e-cigarette use^17^.

Epidemiologic surveillance of e-cigarette use among youth through regular, nationally representative, comparable household surveys helps monitor the changes in the prevalence and patterns of e-cigarette use and provide information to regulate e-cigarette use and evaluate strategies to control use, as appropriate^18^. Yet only about one third of all the countries have comprehensive tobacco use surveillance systems^19^. GYTS provides a feasible, cost-effective, global surveillance method for youth tobacco use through regular school-based surveys^7^. Questions about e-cigarette use introduced in 2014 provides important data for surveillance of e-cigarette use among youth worldwide^18^. A narrative review summarised the prevalence of awareness, ever use, past 30-days use, and regular use up to year 2014^8^ while a systematic review of e-cigarette use among youth aged ≤ 20 years primarily comprising HIC, provides prevalence estimates of ‘ever’, ‘current’ and ‘occasional’ use^20^. A study based on 17 European GYTS sites provides estimates of current e-cigarette use only^21^. Sun et al. reported the prevalence of e-cigarette use in the past 30 days among youth in 68 GYTS countries, while Chan et al.; have also reported e-cigarette use in the past 30 days in 44 GYTS countries. In addition the current literature on e-cigarette use among youth is not comparable across countries because of heterogeneity of measurements and reporting of e-cigarette use^8,22^. To-date GYTS-based reports have not provided estimates of ‘awareness’, ‘ever use’ and have not analysed estimates by e-cigarette regulatory status. As tobacco companies expand their markets beyond HIC, data about e-cigarettes in low-and-middle-income countries (LMIC) are particularly lacking for surveillance purposes. To address this data gap, we report the most comprehensive, comparable country-level estimates to date of e-cigarette awareness, stand-alone e-cigarette use, and ‘dual use’ with conventional cigarettes among youth in 75 countries. We describe the distribution of pooled estimates of e-cigarette awareness, ‘ever e-cigarette use’, ‘current e-cigarette use’, and ‘dual use’ by age, sex, and contextual factors (e-cigarette regulatory policy status, WHO regions, and World Bank income group). We also explored if awareness correlated with ever and current e-cigarette use and if ‘current use’ of e-cigarettes was associated with age, sex, cigarette smoking, and contextual factors.

## Methods

### Design and data sources

We conducted a secondary analysis of the most recent data from 75 GYTS countries/territories that have collected data on e-cigarette use and asked at least one question. This study was conducted in accordance with the Declaration of Helsinki and followed all relevant guidelines and regulations. The detailed methodology of GYTS is available on the US Centers for Disease Control and Prevention (CDC) website. GYTS was reviewed by the National Ethical Committee and parental consent was obtained. GYTS, uses a two-stage cluster sampling method in which schools are selected by probability proportional to their enrolment size. Classes in each selected school are randomly selected until the required sample size was achieved. Students of all ages attending school on the day of the survey answered a standard self-administered core questionnaire with closed ended responses and a set of optional questions adapted by countries according to local needs and priorities. The questionnaires were translated and back translated into the local languages of each country to verify accuracy, and CDC validated and approved the questionnaires at the country level. The core questionnaire covers areas such as tobacco use, exposure to second-hand smoke, attempts to quit smoking, exposure to tobacco product advertising in the media, access to tobacco products, and knowledge, and attitudes towards tobacco use^23^. The number of questions on e-cigarettes in the optional module varied by country and survey year. Countries that included at least one question about ‘awareness’(69/75), ‘ever use’ (54/75) and ‘current use’(67/75) e-cigarettes were included in the analyses.

### Outcome variables

Outcome variables based on questions asked in GYTS were defined as follows; 1) ‘Awareness about e-cigarettes’ was defined as having ever heard about e-cigarettes. 2) ‘Ever e-cigarette use’ was defined as youth who had ever tried or experimented with even one or two puffs or used e-cigarette on at least one day during their lifetime. 3) ‘Current e-cigarette use’ was defined as youth who had used e-cigarette on at least one day during the past 30 days prior to survey date. 4) ‘Current cigarette smoking’ was defined as smoking a cigarette on at least one day during the last 30 days prior to survey date. 6) ‘Dual use’ was defined as youth who had used e-cigarette as well as smoked cigarettes on at least one day over the previous 30 days prior to survey date.

### Explanatory variables

We reviewed the data available on three web resources on regulations about e-cigarette (online supplement 1). E-cigarette regulatory policy status in each country was classified based on the information available from the websites of global tobacco control (https://www.globaltobaccocontrol.org/e-cigarette/domain-classification), global state of harm reduction (https://gsthr.org/countries) and the Tobacco Control Laws (https://www.tobaccocontrollaws.org/legislation). E-cigarette regulatory status for each country was grouped as 1) Most restrictive policies (MRP)- ban of e-cigarettes, 2) Restrictive policies (RP)- regulations on nicotine and/or other contents only, 3) Less restrictive policies (LRP)-allowed selling of e-cigarettes but provided sales restrictions/regulations, and 4) No regulatory policies (NRP)- no reliable information was available^24^. World Bank country classification (based on Gross national income) for the year of the survey was used to classify countries as 1) low, (LIC) 2) lower-middle, (lMIC) 3) upper-middle, (uMIC) and 4) high-income (HIC) using data available at https://datahelpdesk.worldbank.org/knowledgebase/articles/906519-world-bank-country-and-lending-groups (accessed 30/1/2022). GATS countries/territories were grouped according to the WHO regions, namely Africa (AFR), Americas (AMR), Europe (AMR), Eastern Mediterranean (EMR), Western Pacific (WPR) and South-east Asia (SEAR).

### Statistical analyses

Using microdata of each country, weighted prevalence estimates (%) and their 95% confidence intervals (95% CI) were estimated for awareness, ‘ever e-cigarette use’, ‘current e-cigarette use’, and ‘dual use’. The survey weights were adjusted for school, class, and student as per the multistage sampling process of GYTS. The microdata for all 75 countries were combined irrespective of the survey year. On the pooled data set, we calculated pooled estimates of each outcome by age, sex, WHO regions, World Bank income groups, and e-cigarette regulatory status. In the pooled data each survey participant (students) is nested within the school; each school is nested within the country. To account for this multi-level data binary logistic regression models with a random intercept at schools (first level) and country (second level) were used. To explore the factors associated with current e-cigarette use adjusted odds ratios (aOR), their 95% CI, random-effects parameters, and likelihood test ratios were estimated. Age, sex, current cigarette smoking, at the individual level (model 1); e-cigarette regulatory status, and World Bank income group as country-level (model 2) were entered into the models. We explored the relationship of awareness with ever use and current use of e-cigarettes using aggregate data (country-level estimates). We did two-way scatter plots and Spearman’s rank correlation test. Stata version 15/SE (Stata Corp, College Station, Texas, USA) was used for all statistical analyses.

### Ethical approval

This study was based on analyses of publicly available de-identified GYTS data so a separate ethical approval was not required from the author’s institutions.

## Results

### Survey characteristics and sample description

A total of 264, 490 (male = 128,581, female = 135,909) youth surveyed in 75 countries during 2014–2019 was analyzed. Survey samples varied from 163 (Niue) to 9992 (Indonesia). Most GYTS countries were middle income countries (MIC) (uMIC-32, lMIC-22), 20 were HIC. In 23 countries there was no reliable information about e-cigarette regulatory policies (NRP). In 15 countries most restrictive policies (MRP) were in place, 28 countries had restrictive policies (RP), and nine countries had least restrictive policies (LRP). Prevalence of current cigarette smoking among youth was < 3% in six countries, the lowest being in Cambodia (0.9%, 95% CI 0.5, 1.7) and it was > 20% in six countries of which Bulgaria had the highest prevalence (22.8%, 95% CI 18.8, 27.3). Except for seven countries in AMR and EUR, the male-to-female (m-t-f ratio) of current cigarette smoking was > 1 (online supplement 1).

### Country-wise estimates of awareness about and use of e-cigarettes and ‘dual use’

Country-wise prevalence estimates are shown in Table 1. Weighted prevalence of awareness about e-cigarettes in 69 countries varied from 9.4% (95%CI, 7.4, and 11.9) in Cambodia to 95.8% (95% CI 94.8, 96.6) in Poland and it was > 80% in 13 countries most of which were in Europe region (EUR). ‘Ever e-cigarette use’ in 54 countries ranged from 2.3% (95% CI 1.7, 3.1) in Cambodia to 55.1% (95% CI 51.3, 58.9) in Italy. In 7/54 countries, about third to half the youth had ever used e-cigarette. Prevalence of ‘current e-cigarette use’ (67 countries) was lowest in Togo 1.2% (95% CI 0.8, 1.9] and highest in Guam 35.1% [95% CI 32.4, 38.0]. In 30/67 countries, ‘current e-cigarette use’ was > 10%. Prevalence of ‘dual use’ (67 countries) ranged from 0.1% (95% CI 0.0,0.2) in Cambodia to 13.6% (95% CI 12.2, 15.2) in Poland followed by Italy 10.0% (95% CI 8.1,12.2). In 15/67 countries ‘dual use’ was > 5% (Table 2).

**Table 1:** Country-wise overall estimates (%) and their 95% CI of awareness about e-cigarettes, ever and current use of e-cigarettes and dual use for the data available in 75 GYTS countries.

**Country**	Awareness€		Ever use£		Current use¥		Dual®	
**High income**								
Antigua & Barbuda	49.6	[45.9,53.3]	–	–	5.1	[4.1,6.3]	1.0	[0.6,1.5]
Bahrain	60.3	[55.7,64.7]	19.9	[15.8,24.8]	–	–	–	–
Cook Islands	45.2	[45.2,45.2]	16	[16.0,16.0]	9.4	[9.4,9.4]	4.1	[4.1,4.1]
Czech Republic	95.3	[94.4,96.1]	26.2	[23.3,29.3]	11.1	[9.4,13.1]	7.3	[6.0,8.9]
Guam	82.6	[80.5,84.4]	48.2	[44.6,51.8]	35.1	[32.4,38.0]	7.7	[6.4,9.2]
Italy	–	–	55.1	[51.3, 58.9]	18.3	[15.7,21.2]	10	[8.1,12.2]
Kuwait	81.3	[77.3,84.8]	23.6	[19.7,28.0]	–	–	–	–
Latvia	–	–	52.4	[49.0, 55.8]	18.5	[16.6,20.6]	8.6	[7.3,10.2]
Macao	70.9	[67.4,74.1]	–	–	2.7	[1.7,4.3]	0.8	[0.4,1.6]
Niue	71.5	[63.9,78.0]	36.5	[26.9,47.3]	22.5	[15.8,31.0]	8.0	[4.3,14.3]
Oman	44.4	[38.9,50.1]	–	–	6.3	[4.3,9.0]	0.8	[0.5,1.3]
Panama	45.2	[40.0,50.5]	7.7	[6.2,9.5]	6.8	[5.4,8.5]	1.2	[0.7,2.1]
Poland	95.8	[94.8,96.6]	44.5	[42.0,47.1]	26.9	[24.8,29.2]	14	[12.2,15.2]
Qatar	60.9	[56.9,64.7]	17.2	[14.0,20.9]	10.8	[8.6,13.5]	2.1	[1.3,3.2]
San Marino	29.0	[23.6,35.0]	24	[19.3,29.5]	10.7	[7.7,14.6]	4.1	[2.6,6.6]
Seychelles	38.0	[35.0,41.2]	11.9	[10.1,13.9]	7.4	[6.0,9.1]	2.8	[2.0,3.8]
Slovakia	–	–	–	–	8.1	[6.5,9.9]	5.0	[3.8,6.4]
Slovenia	84.9	[82.2,87.3]	16.8	[14.0,20.1]	–	–	0	–
Trinidad & Tobago	71.9	[69.3,74.4]	26.9	[24.1,29.9]	15.7	[13.9,17.8]	3.7	[2.7,4.9]
Uruguay	–	–	25.3	[20.6, 30.1]	14.2	[9.8,20.1]	3.6	[1.5,8.5]
**Upper middle income**	–	–	–	–	–	–	–	
Albania	64.7	[60.3,68.9]	–	–	6.8	[5.8,8.0]	2.4	[1.7,3.5]
Argentina	76.1	[68.2,82.5]	15.7	[11.3,21.3]	8.2	[5.7,11.7]	4.1	[3.0,5.5]
Belarus	81.9	[78.0,85.2]	–	–	–	–	0	–
Belize	33.6	[29.0,38.7]	7.7	[6.0,10.0]	6.3	[5.0,7.9]	2.2	[1.5,3.3]
Bosnia & Herzegovina	90.5	–	–	–	12.1	[10.6, 13.6]	5.8	[4.7, 6.9]
Bulgaria	88.5	[84.8,91.4]	21.6	[19.4,24.0]	10.9	[8.7,13.5]	5.8	[4.2,8.1]
Croatia	91.1	[89.1,92.9]	22.1	[17.8,27.2]	10.8	[8.6,13.6]	6.0	[4.2,8.3]
Cuba	49.7	[44.0,55.5]	–	–	6.0	[4.4,8.2]	2.5	[1.6,3.9]
Dominican Republic	67.3	[62.3,71.8]	20.7	[17.2,24.6]	10.6	[8.5,13.1]	1.7	[1.1,2.5]
Ecuador	50.2	[42.2,58.2]	17.3	[12.0,24.3]	10.8	[8.5,13.5]	3.6	[2.6,4.9]
Fiji	33.9	[31.1,36.9]	12.5	[9.4,16.3]	12.6	[9.9,15.9]	3.2	[2.3,4.5]
Grenada	44.9	[40.4,49.6]	–	–	7.6	[6.1,9.5]	2.1	[1.5,2.9]
Guyana	22.7	[19.8,25.9]	8.0	[5.8,10.8]	8.3	[5.8,11.7]	2.3	[1.5,3.5]
Indonesia	71.3	[68.3,74.1]	22.9	[20.8,25.1]	14.4	[13.0,15.9]	8.5	[7.5,9.6]
Iraq	53.9	[47.8,59.9]	16.1	[12.6,20.3]	9.2	[6.4,13.1]	4.5	[2.6,7.5]
Jamaica	44.0	[44.0,44.0]	16.2	[16.2,16.2]	12.5	[12.5,12.5]	3.9	[3.9,3.9]
Kazakhstan	45.5	[35.3,56.0]	3.4	[2.1,5.7]	2.0	[1.2,3.4]	0.4	[0.2,1.0]
Maldives	58.9	[55.9,61.8]	18.8	[16.5,21.3]			0	
Marshall Islands	28.1	[26.0,30.4]	–	–	18.7	[16.7,21.0]	7.9	[6.8,9.3]
Mauritius	54.2	[47.2,61.0]	–	–	11.3	[8.5,15.0]	5.7	[4.0,8.0]
Macedonia	68.7	[66.0,71.2]	–	–	4.1	[3.3,5.2]	1.7	[1.2,2.3]
Montenegro	81.6	[79.0,83.9]	–	–	–	–	0	–
Paraguay	77.4	[66.3,85.6]	23.3	[18.2, 28.4]	11.3	[9.6,13.2]	1.6	[1.2,2.2]
Peru	52.7	[47.4,58.0]	13.5	[11.8, 15.1]	6.9	[5.7,8.3]	2.3	[1.8,3.0]
Romania	19.6	[17.6,21.7]	14.3	[12.8,15.8]	7.6	[6.5,8.9]	2.9	[2.4,3.6]
Saint Lucia	47.5	[42.4,52.7]	15.7	[13.4,18.3]	10.7	[8.5,13.3]	2.9	[2.1,4.1]
Saint Vincent	39.2	–	–	–	7.5	[6.0, 9.1]	1.9	[1.2,2.6]
Samoa	27.6	[23.8,31.7]	12.2	[9.9,15.0]	12.7	[10.5,15.3]	4.1	[3.1,5.4]
Serbia	–	–	23.4	[22.0, 24.7]	7	[7.0,7.0]	3.4	[3.4,3.4]
Srpska (Is this wrongly spelled?)	86.6	[84.4,88.5]	–	–	–	–	0	–
Suriname	41.3	[36.4,46.5]	–	–	5.7	[4.4,7.3]	2.1	[1.5,3.0]
Thailand	38.6	[32.5,45.0]	5.4	[3.9,7.5]	3.4	[2.5,4.7]	1.8	[1.4,2.5]
**Lower middle income**								
El Salvador	31.5	[27.6,35.7]	8.0	[6.1, 10.0]	3.1	[2.5,3.9]	1.6	[1.2,2.1]
Bolivia	48.6	[44.4,52.9]	12.8	[10.8,15.0]	8.4	[7.2,9.7]	3.1	[2.5,3.8]
Cambodia	9.4	[7.4,11.9]	2.3	[1.7,3.1]	2.4	[1.9,2.9]	0.1	[0.0,0.2]
Congo	37.0	[33.1,41.0]	9.5	[7.1,12.7]	5.5	[3.7,8.2]	1.2	[0.8,1.7]
Georgia	78.9	[73.4,83.5]	–	–	12.4	[9.6,15.9]	3.3	[2.3,4.8]
Ghana	14.3	[11.6,17.4]	8.7	[6.1,12.2]	5.8	[3.9,8.4]	1.2	[0.6,2.2]
Guatemala	39.8	[35.1,44.7]	11.4	[9.7,13.4]	6.1	[4.7,7.7]	3.2	[2.5,4.1]
Kiribati	25.5	[23.4,27.8]	11.7	[9.7, 13.6]	11.2	[9.5,13.3]	4.9	[3.7,6.4]
Kosovo	37.0	[33.6,40.5]	7.6	[6.7,8.5]	4.5	[3.8,5.5]	1.1	[0.8,1.6]
Kyrgyzstan	41.0	[36.4,45.7]	5.3	[4.1, 6.6]	2.9	[2.2,3.9]	0.5	[0.3,0.9]
Lao republic	16.7	[13.9,19.8]	8.6	[7.3, 10.1]	4.9	[3.7,6.3]	1.4	[1.0,1.9]
Mauritania	33.5	[29.7,37.5]	–	–	18.7	[13.8,24.8]	6.7	[4.1,10.6]
Republic of Moldova	87.7	[85.6,89.6]	32.1	[29.3,35.1]	12.8	[11.3,14.5]	3.4	[2.6,4.4]
Mongolia	52.4	[47.5,57.2]	9.9	[8.0,12.0]	3.4	[2.5,4.6]	1.0	[0.6,1.6]
Morocco	44.1	[38.1,50.2]	–	–	5.4	[4.0,7.3]	0.8	[0.5,1.3]
Nicaragua	–	–	14.5	[12.2, 16.9]	8.6	[7.7,9.6]	3.1	[2.7,3.7]
Papua New Guinea	20.4	[18.3,22.8]	–	–	18	[15.0,21.3]	7.4	[5.6,9.7]
Philippines	43.3	[39.1,47.6]	12.1	[10.0,14.5]	–	–	0	–
Tunisia	54.6	[50.5,58.7]	6.4	[5.3,7.6]	4.8	[3.9,6.0]	1.7	[1.1,2.5]
Ukraine	93.2	[91.1,94.8]	36.3	[32.8,40.0]	17.4	[14.8,20.4]	3.7	[2.6,5.3]
Vanuatu	21.3	[18.4,24.5]	10.7	[8.4,13.6]	7.2	[5.5,9.4]	4.7	[3.4,6.4]
Yemen	29.5	[23.7,36.1]	14.4	[12.1,17.1]	13.9	[11.8,16.4]	2.3	[1.5,3.5]
**Low income**								
Togo	16.3	[11.3,22.9]	–	–	1.2	[0.8,1.9]	0.4	[0.2,0.8]

€ -69 countries, £- 54 countries, ¥- 67 countries, ®-67 countries.

**Table 2:** Pooled estimates and their 95% CI of awareness about e-cigarette, ever and current e-cigarette use, and ‘dual use’ among the youth by their age, sex, and contextual factors (WHO regions, World Bank taxonomy, and regulatory policy status).

	Awareness		Ever use		Current use		Dual use	
	P (%)	95% CI	P (%)	95% CI	P (%)	95% CI	P (%)	95% CI
Total	56.7	[55.2,58.2]	20.2	[19.2,21.3]	10.9	[10.3,11.6]	4.6	[4.1,5.1]
**Age**								
≤ 11 years*	53.2	[47.7,58.7]	25	[19.9,31.0]	17.6	[13.6,22.5]	10.3	[7.1,14.8]
12 years	49.9	[46.9,53.0]	16.1	[13.9,18.6]	8.7	[7.2,10.5]	3.0	[2.2,4.0]
13 years	52.4	[50.0,54.8]	16.1	[14.7,17.7]	8.3	[7.5,9.2]	3.1	[2.6,3.7]
14 years	57.0	[55.0,58.9]	19.9	[18.4,21.6]	10.8	[9.9,11.8]	4.4	[3.8,5.2]
15 years	57.6	[55.4,59.8]	21.7	[20.3,23.3]	11.1	[10.2,12.1]	4.6	[4.1,5.3]
16 years	63.3	[60.6,65.9]	24.9	[23.2,26.8]	14.2	[12.7,15.7]	6.6	[5.7,7.5]
≥ 17 years*	63.8	[60.3,67.1]	24.8	[22.8,27.0]	14.6	[12.9,16.4]	7.3	[5.8,9.2]
**Sex**								
Male	61.5	[59.9,63.1]	28.5	[26.8,30.3]	16	[14.8,17.3]	7.6	[6.7,8.7]
Female	51.9	[50.1,53.6]	11.8	[10.8,12.8]	5.6	[5.2,6.1]	1.6	[1.4,1.8]
**WHO regions (number of countries)**								
AFR (6)	19.1	[16.9,21.5]	8.8	[6.4,12.0]	5.7	[4.4,7.5]	1.4	[1.0,2.0]
AMR (21)	58.1	[55.4,60.8]	18.7	[16.6,21.0]	8.2	[7.4,9.1]	2.8	[2.5,3.1]
EMR (8)	46.4	[43.8,49.0]	14.3	[12.4,16.3]	8.2	[7.1,9.5]	2.2	[1.6,3.2]
SEAR (3)	66.9	[64.4,69.3]	20.5	[18.7,22.5]	12.9	[11.7,14.3]	7.6	[6.7,8.5]
WPR (14)	38.2	[34.7,41.8]	15.3	[13.5,17.2]	4.4	[3.9,4.9]	0.2	[0.2,0.3]
EUR (23)	74.6	[72.1,76.9]	34.5	[32.5,36.5]	15.5	[14.4,16.6]	6.7	[6.0,7.3]
**World Bank taxonomy (number of countries)**								
Low (1)	16.3	[11.3,22.9]	–	–	1.2	[0.8,1.9]	0.4	[0.2,0.8]
Lower Middle (12)	40.6	[38.6,42.7]	16.5	[15.3,17.8]	7.6	[6.9,8.3]	1.3	[1.1,1.5]
Upper Middle (32)	64.0	[62.1,65.9]	19.4	[18.0,20.9]	11.2	[10.3,12.2]	5.8	[5.1,6.5]
High (20)	83.6	[82.3,84.8]	39.4	[37.0,41.8]	18.5	[17.2,20.0]	9.0	[8.0,10.0]
**E-cigarette regulatory policy status (number of countries)**								
NRP (23)	46.2	[43.1,49.4]	13.2	[10.9,15.9]	6.8	[6.0,7.7]	1.6	[1.3,1.9]
LRP (9)	48.5	[44.7,52.4]	19.9	[17.6,22.5]	15.4	[13.4,17.5]	3.1	[2.3,4.0]
RP ((28)	65.2	[63.0,67.4]	22.4	[21.0,23.8]	13.4	[12.4,14.5]	6.9	[6.2,7.6]
MRP (15)	47.5	[42.1,53.0]	17.2	[14.9,19.8]	5.6	[4.6,6.7]	2.4	[1.9,2.9]

All bivariate comparisons were statistically significant p < 0.001 by Chi-square statistic. *GYTS questionnaire did not collect exact age of those aged ≤ 11 years and ≥ 17 years.

### Pooled prevalence estimates of awareness, e-cigarette use, and dual use and their distribution

Pooled prevalence estimate, overall and by age, sex, WHO regions, World Bank taxonomy, and e-cigarette regulatory policy status are shown in Table 2. Overall pooled estimates were 56.7% (95% CI 55.2, 58.2) for awareness of e-cigarette, the estimates for ‘ever use’, current use’, and ‘dual use’ were 20.2%, (95% CI 55.2, 58.2), 10.9% (95% CI 19.2, 21.3) and 4.6% (95% CI 4.1, 5.1) respectively. Youth from EUR (74.6%) and HIC (83.6%) had the highest e-cigarette awareness. Boys, higher age, and youth from countries with RP had a higher e-cigarette awareness. A similar pattern was observed for ‘ever’ use’, and ‘current use’ of e-cigarettes and ‘dual use’. Current e-cigarette use was also threefold higher (1.6% vs. 5.6%) while ‘dual use’ was fivefold higher (7.6% vs. 1.6%) among boys compared to girls, and prevalence also increased with age (Table 3). Notably, among those aged 11 years and less, ever (25.0%) and current (17.6%) e-cigarette use, and ‘dual use’ (10.3%) was higher than in the other ages. On bivariate comparisons, all the differences in pooled estimates were statistically significant (*p* < 0.001).

**Table 3:** Socio-demographic and contextual factors associated with current e-cigarette use among youth by multi-level regression analyses.

	Univariate analyses		Multi-level model 1		Multi-level model 2	
	Crude OR (95% CI)		Adj. OR (95% CI)		Adj. OR (95% CI)	
**Age groups (in** years)						
11–13	1	< 0.001	1	< 0.001	1	< 0.001
14	1.3 (1.1, 1.4)		1.2 (1.2, 1.3)		1.2 (1.2, 1.3)	
15	1.3 (1.1, 1.5)		1.3 (1.2, 1.34)		1.3 (1.2, 1.3)	
≥ 16	1.7 (1.49, 2.0.)		1.3 (1.3, 1.4)		1.3 (1.2, 1.4)	
**Sex**						
Female	1		1	< 0.001	1	< 0.001
Male	3.2 (2.9, 3.6)	< 0.001	1.7 (1.6, 1.7)		1.7 (1.6, 1.7)	
**Current cigarette smoker**						
No	1		1	< 0.001	1	< 0.001
Yes	12.4 (11.2, 13.7)	< 0.001	9.9 (9.6,10.4)		9.9 (9.5, 10.3)	
**Contextual factors**						
**E-cigarette regulatory policy status**						
NRP	1				1	
LRP	2.5 (2.0, 3.1)	< 0.001			0.9 (0.8, 1.1)	0.401
RP	2.2 (1.8, 2.5)	< 0.001			1.8 (1.6, 2.0)	< 0.001
MRP	0.8 (0.6, 1.0)	0.069			0.6 (0.6, 0.7)	< 0.001
**World Bank taxonomy**						
lMIC	1				1	
uMIC	1.6 (1.3, 1.8)	< 0.001			1.1 (1.0, 1.2)	0.011
HIC	2.8 (2.4, 3.2)	< 0.001			2.4 (2.2, 2.7)	< 0.001
**Random-effects parameters**						
School level	SD		0.00000003		0.00000002	
	SE		0.000664		0.0004885	
Country level	SD		0.8746556		0.6879336	
	SE		0.208748		0.0182421	
**Model comparison**						
Likelihood	Chi-square statistic		4384.7		2552.8	
Ratio test^a^	*P* value		0.0000		0.0000	

^a^Based on the results on likelihood ratio tests, estimates of multilevel logistic regression were preferred than fixed effect models.

SD-standard deviation, SE-standard error.

*Ages 11–13 and ≥ 16 were merged owing to small numbers.

### Sex-differentials

Sex-wise estimates in each country are shown in online supplement 2. Country-wise, in nearly all countries male-to-female (m-t-f) ratio for e-cigarette use was marginally above 1. However, both ‘ever e-cigarette use’, and ‘current e-cigarette use’ m-t-f ratio was > 1 in most countries the largest m-t-f ratio being 6.7 for ‘ever use’ (Indonesia) and 6.8 for ‘current use’ (Qatar). The m-t-f ratio for ‘dual use’ was diverse. It was < 1 in four countries (Ghana, Mauritania, Paraguay, and Slovenia), and among the countries with a magnitude of m-t-f ratio > 1 the highest ratio was in Morocco (31.0) and Qatar (28.2). Generally, the magnitude of the m-t-f ratio (> 1) for ‘current cigarette smoking’ (online supplement 1) was higher than that for ‘current e-cigarette use’. However, in 25 countries (mainly in 10 countries of EUR) m-t-f ratio for ‘current e-cigarette’ use was larger than that of current cigarette smoking. Notably, in some SEAR and EMR countries, the m-t-f ratio was > 1 but the magnitude of m-t-f ratio for e-cigarette use was smaller than that for cigarette smoking. In Uruguay, Antigua and Barbuda, and Argentina, the m-t-f ratio for e-cigarette were > 1 for e-cigarette and < 1 for cigarette smoking (online supplement 2).

### Factors associated with e-cigarette use

**Figure 1:**
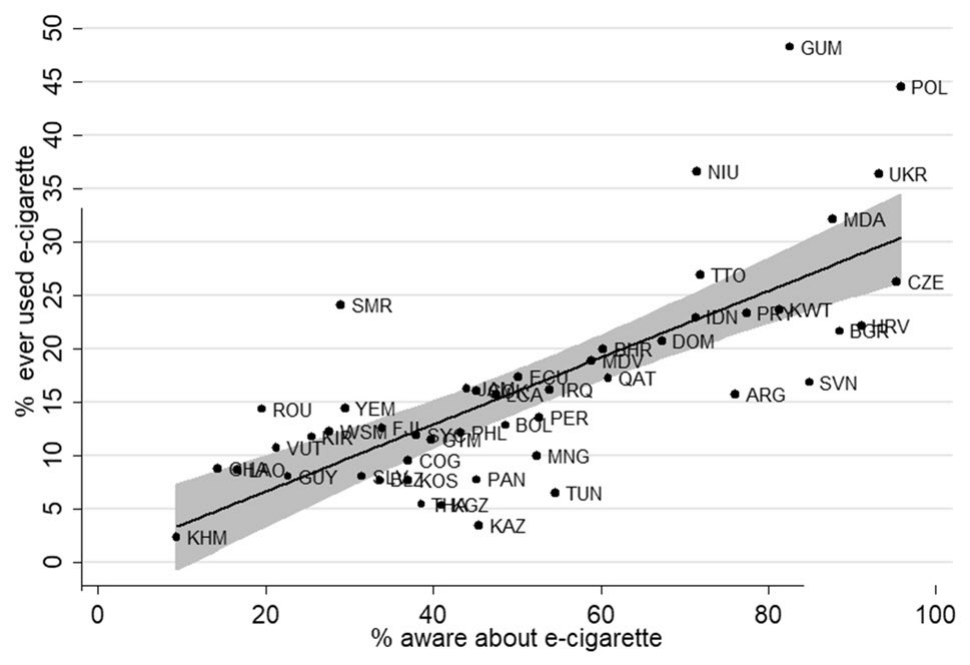
Two-way scatter plots between the prevalence of awareness and ever e-cigarette use (both sexes) in 49 countries. Cambodia- KHM, Ghana-GHA, Lao republic-LAO, Romania-ROU, Vanuatu-VUT, Guyana-GUY, Kiribati-KIR, Samoa-WSM, San Marino-SMR, Yemen-YEM, El Salvador-SLV, Belize-BLZ, Fiji-FJI, Kosovo-KOS, Congo-COG,Seychelles-SYC,Thailand-THA,Guatemala-GTM,Kyrgystan-KGZ,Philippines-PHL,Jamaica-JAM,Panama-PAN,Cook islands-COK, Kazakhstan-KAZ, Saint Lucia-LCA,Bolivia-BOL,Ecuador-ECU,Mongolia-MNG,Peru-PER,Iraq-IRQ, Tunisia-TUN, Maldives-MDV, Bahrain-BHR, Qatar-QAT, Dominican Republic-DOM, Indonesia-IDN, Niue-NIU, Trinidad and Tobago-TTO, Argentina-ARG, Paraguay-PRY, Kuwait-KWT, Guam-GUM, Slovenia-SVN, Republic of Moldova-MDA, Bulgaria-BGR, Croatia-HRV, Ukraine-UKR, Czech republic-CZE, Poland-POL. (Spacing after the comma should be consistently applied).

**Figure 2:**
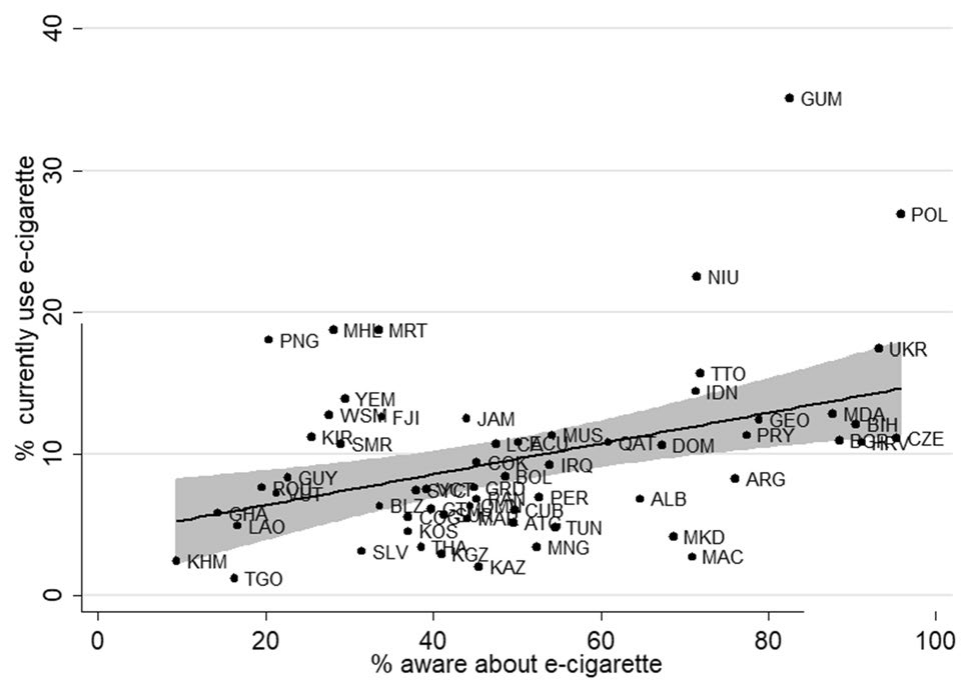
Two-way scatter plots between the prevalence of awareness and current EC use (both sexes) in 61countries. Congo-COG, Ghana-GHA, Mauritania-MRT, Mauritius-MUS, Seychelles-SYC, Togo-TGO, Antigua and Barbuda-ATG, Argentina-ARG, Belize-BLZ, Bolivia-BOL, Cuba-CUB, Dominican republic-DOM, Ecuador-ECU, El Salvador-SLV, Grenada-GRD, Guatemala-GTM, Guyana-GUY, Jamaica-JAM, Panama-PAN, Paraguay-PRY, Peru-PER, Saint Lucia-LCA, Suriname-SUR, Trinidad and Tobago-TTO, Yemen-YEM, Iraq-IRQ,Morocco-MAR,Oman-OMN,Qatar-QAT,Tunisia-TUN,Thailand-THA,Indonesia-IDN,Vanuatu-VUT,Cambodia-KHM, Cook islands-COK, Fiji-FJI, Guam-GUM, Kiribati-KIR, Lao republic-LAO, Macao-MAC, Marshall islands-MHL, Mongolia-MNG, Niue-NIU, Papua new guinea-PNG, Samoa-WSM, Ukraine-UKR, Albania-ALB, Bulgaria-BGR, Croatia-HRV, Czech republic-CZE, Georgia-GEO, Kazakhstan-KAZ, Kosovo-KOS, Kyrgyzstan-KGZ, Macedonia-MKD, Poland-POL, Moldova, Republic of-MDA, Romania-ROU, San Marino-SMR, Bosnia-BIH, Saint Vincent and The Grenadines-VCT. (Spacing after the comma should be consistently applied).

Awareness about e-cigarettes was positively correlated with ‘ever use’ (r = 0.731, (95%CI 0.566 to 0.839, *p* < 0.001) (Fig. 1) as well as ‘current use’ (r = 0.333, 95% CI 0.089 to 0.540, *p* = 0.009) (Fig. 2). The correlations were consistent and statistically significant among boys (r = 0.717, 95% CI 0.546 to 0.831, *p* < 0.001 and r = 0.347 95% CI 0.097 to 0.555, *p* = 0.008) and girls (r = 0.549, 95% CI 0.319 to 0.718, *p* < 0.001 and r = 0.281 95% CI 0.027 to 0.501, *p* = 0.031). Higher age, being a boy, current cigarette smoking, higher country income and restrictive e-cigarette regulations had higher odds of being a ‘current e-cigarette user’. For instance, after adjusting for other factors, boys had about 1.7 times higher odds of being a current e-cigarette user (aOR 1.7 95%CI 1.6, 1.7), and youth who currently smoked cigarettes had 10 times higher odds of being current e-cigarette users (aOR 9.9, 95% CI 9.5, 10.3). Youth from a HIC compared to lMIC and a country having restrictive e-cigarette regulations compared to NRP had 2.4 times (aOR 2.4, 95% CI 2.2, 2.7) and 1.8 times (aOR 1.8, 95% CI 1.6, 2.0) higher odds of being current e-cigarette users  respectively (Table 3) respectively. Youth in countries with most restrictive e-cigarette regulations compared to NRP had 0.6 times lower odds of being current e-cigarette users (aOR 0.6, 95% CI 0.6, 0.7).

## Discussion

Amid growing concern about increasing e-cigarette use among the youth particularly in HIC, and a relative scarcity of data for LMIC, our report provides the most up-to-date reliable estimates using comparable GYTS data. Our analyses of GYTS microdata showed that nearly half of the youth are aware of e-cigarettes and one third of them have used e-cigarette in their lifetime. Stand-alone e-cigarettes use and ‘dual use’ were several times higher among boys than girls. E-cigarette use was high among the youth in European countries and HIC. In some countries, up to half of the youth had used an e-cigarette in their lifetime and one third to one quarter of them were current e-cigarette users. In countries with a higher prevalence of e-cigarette use ‘dual use’ was common.

### Strengths and limitations of this study

A standardized survey methodology, questionnaire and acceptable response rates in GYTS provide comparable estimates^25^. Compared with the current literature^8,18,20,21^, we provide estimates for more uMIC and lMIC in which regulatory policies are weak or absent^26^. Our study is the first to report on the distribution of e-cigarette use among the youth by age, sex, country income groups, and e-cigarette regulatory status to inform public health policy and assess the impact of existing regulations^27^. Existing reports on estimates of e-cigarette use only report about ever, current, and occasional e-cigarette use^20,21^. However this report provides estimates of e-cigarette awareness and ‘dual use’. Several limitations should be considered while interpreting our results. Self-reported behaviors are subject to reporting bias particularly in middle-income countries. Regardless, current use on one or more days in the 30 days before the survey date is known to reduce information bias and is a reliable indicator of current use^28^. Our pooled estimates do not represent global prevalence or prevalence estimates for World Bank income groups and WHO regions. The number of countries in which GYTS included optional questions on e-cigarette use was smaller than the number of countries covered by GYTS. The question on e-cigarette use did not distinguish between electronic nicotine delivery systems (ENDS) and electronic non-nicotine delivery systems (ENNDS)^23^. Therefore, we were unable to provide estimates for these two types i.e. ENDS and ENNDS^29^. Our estimates for 2014 to 2019, are not very up-to-date because e-cigarette use patterns among youth are evolving rapidly due to aggressive marketing strategies by tobacco companies.

### Interpretation and comparison with related studies

Our findings are consistent with published reports that e-cigarette use varies across countries^8,17,21^, based on the different indicators used to define e-cigarette use^22^. The cross national difference in e-cigarette use are due to the different regulatory frameworks for e-cigarettes^26,30^. The very high prevalence of awareness is consistent with studies from HIC in which exposure to e-cigarettes through online media^31,32^. or witnessing of e-cigarette use by youth among peers, family, or in public is common. A significant correlation between estimates of awareness and ever use of e-cigarette use supports the argument that increased awareness through aggressive advertising and marketing has led to experimentation with e-cigarette (to try them out) and subsequent regular use^33^. Higher rates of e-cigarette use in HIC may be attributed to higher disposable income among youth compared to their peers in uMIC or lMIC^34^. Higher pocket money among the youth led to higher odds of them being e-cigarette use in a European study^21^. Tobacco company strategies such as advertising attractive flavors, sleek models, and online sales have increased e-cigarette use in HIC^21,35^ however such data are lacking for uMIC and lMIC. The distribution of pooled estimates by WHO region is consistent with existing reports from the EUR and AMR^8,20,21,36^.

Higher e-cigarette use rates among boys than girls, is comparable to sex difference reported about cigarette smoking^37^. The ratio of boys to girls for cigarette smoking > 1 is consistent with the existing literature^7,37^. In most countries the difference between boys and girls is greater for e-cigarette use compared to cigarette smoking. In some EMR countries and SEA, where female smoking is culturally unacceptable^38^. The difference was narrow i.e. m-t-f for e-cigarette indicates that e-cigarette use among girls may be higher. The higher rates of e-cigarette use among 11–13 years old suggests that the age at initiation for e-cigarette use was lower^5^ as in Europe and America^21,39^. The results suggest that experimentation with e-cigarette occur at a very young age, which warrants stricter policies regarding the minimum age for e-cigarette purchase^39,40^. Evidence from a few countries suggests that ‘dual use’ of cigarette smoking and e-cigarette use among the youth is on the rise^13,14^. There is growing evidence that e-cigarette use at younger ages leads to subsequent initiation of cigarette smoking^9,41^ and a lifelong nicotine dependence^42^. Exposing youth to nicotine at a young age is a ploy by tobacco companies to entice them to smoke cigarettes^33^. However, in the absence of detailed information we cannot preclude if the youth had initiated cigarette smoking or e-cigarette or switched between these during the previous 30 days. Adult smokers have been reported to use e-cigarettes for cigarette smoking cessation^43^. E-cigarette questions included in GYTS did not inquire about the purpose of e-cigarette use as youth are unlikely to be using e-cigarettes to quit smoking^44^.

### Implications of the results on policy and future research

Understanding the distribution of e-cigarette use across these socio-demographic groups is useful for policy-making on e-cigarette regulations^27^. Our results provide deeper insights into the age and sex distribution of e-cigarette use among youth to assist formulation of age-gender-specific e-cigarette regulatory policies^37^. However, we were unable to examine the distribution of e-cigarette use by youth’s income status because GYTS does not gather such data consistently across countries. However, the results of previous studies indicate that higher income is a determinant of e-cigarette use among youth and adults^21,45^. Our report showed that e-cigarette use is substantial among the youth in uMIC countries not just in HIC. Our findings call for continued monitoring at HIC and expansion of monitoring to additional uMIC and lMIC countries for surveillance using standardized survey methods and indicators of e-cigarette use^46^. GYTS results presented in this report could serve as a benchmark for future surveillance. Although surveys typically collect data on types of e-cigarette (ENDS/ENNDS), flavors and designs, surveillance should cover basic indicators of e-cigarette use behaviors covering recommended indicators^46^.

## Conclusion

Prevalence of e-cigarette use among the youth varied by income groups and WHO regions in World bank countries. E-cigarette was highest in HIC and those in EUR and AMR regions. To better understand the determinants of e-cigarette use and formulate future e-cigarette regulatory policies, an expanded and standardized surveillance system is needed. E-cigarettes need to be regulated in the MIC to prevent escalation of its use as in HIC.

## Supplementary Information

Supplementary Information 1.

Supplementary Information 2.

## Data Availability

The datasets used and/or analysed during the current study available on the website of Centre for Disease Control, Atlanta, USA. The data sets and Stata code used for analyses are available from the corresponding author on reasonable request.
