# Supplementary material for: Electronic cigarettes use and ‘dual use’ among the youth in 75 countries: estimates from Global Youth Tobacco Surveys (2014–2019)
Source: Sci Rep. 2022 Dec 5;12:20967. doi: 10.1038/s41598-022-25594-4 (PMC9722706; doi:10.1038/s41598-022-25594-4)
Supplement: Supplementary file 1 — Supplementary Information 1. [file 41598_2022_25594_MOESM1_ESM.pdf]

eTable 1

Survey characteristics, cigarette smoking prevalence, e-cigarette regulatory status, and World Bank classification of the 75 GYTS countries.

Country	Survey year	Response rate*	Current cigarette smoking		Sample size		EC regulation	World Bank category
			Prevalence (95% CI)	m-t-f ratio	Total sample	m-t-f ratio		
**AFRICA region (AFR)**								
Congo	2019	-	4.3 [3.4,5.5]	2.2	6396	1	NRP	LM
Ghana	2017	93.8	3.0 [2.0,4.5]	1.2	5664	0.9	NRP	LM
Mauritania	2018	91.3	13.2 [9.1,18.6]	1.1	3,740	0.8	NRP	LM
Mauritius	2016	89.4	15.2 [11.3,20.0]	3.2	4,141	0.9	MRP	UM
Seychelles	2015	79.8	15.6 [13.4,18.2]	1.8	2,485	0.9	MRP	H
Togo	2019	-	3.2 [2.4,4.3]	7.6	3,917	1.1	RP	L
**REGION OF THE AMERICAS (AMR)**								
Uruguay	2019	68.8	9.6 [6.5,13.8]	0.6	2,355	1	MRP	H
Antigua & Barbuda	2017	87.1	2.2 [1.6,3.1]	0.7	2,268	1.1	MRP	H
Argentina	2018	61.3	19.1 [13.2,26.8]	0.8	1,453	1.3	MRP	UM
Belize	2014	83.6	8.1 [6.1,10.6]	2	1,900	0.8	RP	UM
Bolivia	2018	80.0	9.4 [8.0,11.0]	1.9	5,155	1.1	RP	LM
Cuba	2018	93.1	10.1 [7.0,14.5]	1.4	4,172	1	NRP	UM
Dominican Republic	2016	79.5	4.5 [3.1,6.4]	1.2	1,532	0.8	NRP	UM
Ecuador	2016	83.9	9.6 [7.9,11.6]	1.6	5,462	1	RP	UM
El Salvador	2015	82.7	11.5 [9.8,13.4]	1.7	3,215	0.9	RP	LM
Grenada	2016	91.8	6.6 [5.1,8.5]	1.8	2,133	1	RP	UM
Guatemala	2015	81.3	13.5 [11.4,16.0]	1.4	4,301	1.1	RP	LM
Guyana	2015	79.6	6.7 [4.8,9.4]	3.3	1,697	0.8	RP	UM
Jamaica	2017	56.7	12.3 [12.3,12.3]	1.2	1,685	0.8	RP	UM
Panama	2017	83.6	4.2 [3.1,5.7]	1.4	2,621	1	MRP	H
Paraguay	2019	-	2.8 [2.2,3.5]	1	4,698	0.9	RP	UM
Peru	2019	-	6.4 [5.1,8.0]	1.7	4,148	1	NRP	UM
Saint Lucia	2017	84.3	6.2 [5.0,7.7]	1.5	1,495	0.9	RP	UM
Saint Vincent	2018	-	6.3 [4.7,8.0]	1.3	1,519	0.9	NRP	UM
Suriname	2016	86.2	10.5 [8.0,13.8]	2.4	2,212	0.9	MRP	UM
Trinidad &Tobago	2017	90.7	7.5 [5.8,9.7]	2	4,128	0.8	RP	H
Nicaragua	2019	-	12.3 [10.7,14.1]	1.8	8,735	0.9	MRP	LM

**EASTERN MEDITERRANEAN REGION (EMR)**								
Yemen	2014	85.1	6.8 [5.0,9.4]	3	2,107	1.1	RP	LM
Bahrain	2015	76.9	10.8 [7.7,14.9]	4	3,641	1	NRP	H
Iraq	2019	89.0	13.2 [9.3,18.5]	3.1	2,560	2.3	RP	UM
Kuwait	2016	87.7	13.5 [10.6,17.1]	3.9	2,477	0.8	MRP	H
Morocco	2016	86.0	2.6 [1.8,3.7]	4	3,915	1	NRP	LM
Oman	2016	91.9	2.6 [1.6,4.2]	4.9	2,208	0.9	MRP	H
Qatar	2018	89.0	6.7 [4.7,9.4]	4.1	2,071	1	MRP	H
Tunisia	2017	92.8	7.9 [6.4,9.7]	11.2	2,448	0.8	RP	LM
**SOUTH-EAST ASIAN REGION (SEAR)**								
Thailand	2015	86.1	10.9 [8.2,14.4]	3.5	1,876	0.8	MRP	UM
Indonesia	2019	91.0	19.6 [17.8,21.6]	17.8	9,992	0.8	RP	UM
Maldives	2019	70.0	5.3 [4.3,6.5]	3.4	4,799	0.8	RP	UM
**WESTERN PACIFIC REGION (WPR)**								
Vanuatu	2017	64.5	21.2 [17.4,25.7]	1.9	2,257	0.7	NRP	LM
Cambodia	2016	81.0	0.9 [0.5,1.7]	5	3,716	0.8	MRP	LM
Cook Islands	2016	56.6	19.1 [19.1,19.1]	1.6	614	0.9	NRP	H
Fiji	2016	80.7	12.1 [9.2,15.7]	2.1	3,697	0.8	RP	UM
Guam	2017	70.7	11 [9.5,12.7]	1.6	2,506	1	RP	H
Kiribati	2018	71.2	24.1 [21.1,27.3]	2.3	2,622	0.8	NRP	LM
Lao republic	2016	98.0	7.9 [6.5,9.6]	6.7	6,550	1	MRP	LM
Macao	2015	76.6	4.8 [2.7,8.4]	1.8	1,907	1	MRP	H
Marshall Islands	2016	83.8	22 [19.5,24.8]	3.2	3,522	0.8	NRP	UM
Mongolia	2019	92.1	4.7 [3.6,6.0]	8.3	4,146	0.9	NRP	LM
Niue	2019	77.3	11.8 [6.9,19.4]	2.3	163	0.9	RP	H
Papua New Guinea	2016	60.0	26.2 [22.9,29.8]	1.9	2,301	1	RP	LM
Philippines	2015	82.7	14.7 [12.9,16.7]	2.9	8,789	0.8	LRP	LM
Samoa	2017	61.9	12.6 [9.9,15.9]	4.2	2,076	0.6	NRP	UM
**EUROPEAN REGION (EUR)**								
Ukraine	2017	81.6	7.7 [5.8,10.1]	1.3	4,065	1	RP	LM
Albania	2015	89.3	8.4 [6.2,11.3]	2.7	4,672	0.9	LRP	UM
Belarus	2015	81.3	7.8 [6.3,9.5]	1	2,993	1	NRP	UM
Bosnia & Herzegovina	2019	-	17.4 [14.4,20.4]	1.4	5,483	1.1	NRP	UM
Bulgaria	2015	86.0	22.8 [18.8,27.3]	0.8	4,042	0.9	LRP	UM

Croatia	2016	95.8	16.7 [12.9,21.3]	1.1	3,250	1.1	LRP	UM
Czech Republic	2016	78.3	15.2 [12.9,17.9]	0.9	3,926	1	LRP	H
Georgia	2017	78.7	8.3 [6.0,11.4]	3.1	1,345	1	RP	LM
Italy	2018	-	21 [17.9,24.4]	0.7	1,680	1	LRP	H
Kazakhstan	2014	96.2	1.8 [1.1,3.0]	1.5	2,083	1.1	NRP	UM
Kosovo	2016	94.6	3.8 [2.9,4.9]	3.1	5,031	1	NRP	LM
Kyrgyzstan	2019	88.8	2.7 [1.8,4.0]	5	6,145	1	NRP	LM
Latvia	2019	-	15.7 [13.2,18.7]	1	4,226	1.1	LRP	H
Macedonia	2016	92	8.1 [6.5,10.1]	1.5	5,141	1	NRP	UM
Montenegro	2018	92.6	6.2 [5.1,7.5]	1.2	4,216	1	RP	UM
Poland	2016	81.7	20.5 [18.6,22.6]	1.1	5,154	0.9	RP	H
Republic of Moldova	2019	93.3	7.8 [6.4,9.4]	2.6	4,717	1	RP	LM
Romania	2017	-	7.7 [6.5,9.1]	1.4	5,409	0.9	RP	UM
San Marino	2018	92	8.3 [5.6,12.2]	1.1	624	1.2	NRP	H
Serbia	2017	-	13.7 [13.7,13.7]	1	3,861	1	RP	UM
Slovakia	2016	81.7	17.3 [15.0,19.9]	0.9	3,997	1	LRP	H
Slovenia	2017	68.0	9.5 [6.8,13.2]	0.8	2,629	0.9	LRP	H
Srpska	2018	-	12 [9.8,14.8]	1.3	5,346	0.9	NRP	UM

* data on response rate was obtained from the country reports (-indicates unavailability of the country report)
