# Supplementary material for: Electronic cigarettes use and ‘dual use’ among the youth in 75 countries: estimates from Global Youth Tobacco Surveys (2014–2019)
Source: Sci Rep. 2022 Dec 5;12:20967. doi: 10.1038/s41598-022-25594-4 (PMC9722706; doi:10.1038/s41598-022-25594-4)
Supplement: Supplementary file 2 — Supplementary Information 2. [file 41598_2022_25594_MOESM2_ESM.pdf]

e-Table, Sex-wise prevalence estimates of awareness, e-cigarette use, and dual-use in 75 countries

	Boys								Girls							
**Country**	**awareness**		**Ever use**		**Current use**		**Dual-use**		**Awareness**		**Ever use**		**Current use**		**Dual-use**	
Seychelles	44	[40.4,47.7]	15.6	[13.2,18.3]	10.2	[8.1,12.8]	4.2	[2.9,6.0]	32.2	[28.5,36.1]	8.1	[6.1,10.6]	4.6	[3.4,6.2]	1.4	[0.8,2.3]
Uruguay			26.9	[18.8, 34.9]	18	[10.7,28.7]	4.4	[1.1,15.8]					9.7	[7.9,11.9]	2.7	[1.9,3.9]
Antigua and Barbuda	48.9	[44.6,53.1]			5.2	[4.0,6.8]	0.6	[0.3,1.3]	50.3	[45.6,55.1]			4.3	[3.1,6.1]	0.8	[0.4,1.6]
Panama	48.2	[42.7,53.7]	7.9	[6.2,10.0]	8	[6.2,10.2]	1.5	[0.9,2.7]	42.1	[36.5,47.9]	7.1	[5.6,9.0]	5.3	[3.9,7.2]	0.9	[0.5,1.6]
Trinidad and Tobago	72.3	[68.8,75.5]	31.8	[28.8,34.9]	19.8	[17.2,22.6]	5.1	[3.7,7.1]	71.6	[68.9,74.2]	22.4	[19.3,25.9]	12	[10.0,14.4]	2.3	[1.6,3.3]
Bahrain	68.2	[63.5,72.5]	29	[24.1,34.6]			0		53	[47.8,58.0]	11.4	[9.9,13.0]			0	
Kuwait	85.7	[81.1,89.3]	33.2	[28.4,38.4]			0		77.5	[71.8,82.3]	14.9	[12.4,17.8]			0	
Oman	49.1	[43.9,54.3]			9.7	[7.2,13.0]	1.4	[0.9,2.0]	39.6	[31.1,48.8]			2.5	[1.5,4.3]	0.2	[0.1,0.8]
Qatar	66.1	[61.4,70.4]	23	[18.8,27.9]	14.8	[11.8,18.2]	3.4	[2.2,5.2]	56.2	[51.2,61.1]	12	[9.6,14.9]	7.2	[5.3,9.7]	0.8	[0.4,1.6]
Cook Islands	45.1	[45.1,45.1]	16.5	[16.5,16.5]	10.2	[10.2,10.2]	5	[5.0,5.0]	45.4	[45.4,45.4]	15.4	[15.4,15.4]	8.3	[8.3,8.3]	3.1	[3.1,3.1]
Guam	83.4	[80.5,85.9]	51.1	[47.0,55.2]	38.7	[35.2,42.3]	9.4	[7.6,11.5]	81.9	[79.1,84.4]	44.2	[40.2,48.3]	30.4	[27.3,33.7]	5.3	[4.0,7.0]
Macau	70.9	[66.6,74.9]			4	[2.4,6.6]	1.1	[0.4,2.7]	70.7	[66.5,74.6]			1.3	[0.7,2.6]	0.6	[0.3,1.1]
Niue	79.9	[69.3,87.5]	46	[34.1,58.5]	29.8	[20.4,41.3]	12.9	[6.5,24.0]	65.3	[54.2,75.0]	27.7	[17.5,40.9]	16.7	[9.2,28.5]	3.8	[1.3,10.1]
Czech Republic	95.5	[94.3,96.4]	27.3	[23.7,31.2]	12.4	[10.1,15.3]	7.9	[6.0,10.2]	95.1	[93.8,96.2]	25	[21.7,28.7]	9.7	[8.0,11.8]	6.7	[5.1,8.7]
Italy			47.2	[42.1, 52.2]		[18.8,26.7]	11.5	[8.9,14.7]			42.5	[38.0, 47.0]		[11.3,16.3]	8.3	[6.6,10.4]
Latvia			58.7	[55.2, 62.1]		[20.5,25.7]	10	[8.3,11.9]			45.8	[41.4, 50.2]	13.9	[11.6,16.5]	7.2	[5.7,9.1]
Poland	94.9	[93.2,96.3]	48	[45.2,50.7]	31.5	[28.8,34.4]	15.3	[13.4,17.3]	96.8	[95.9,97.5]	40.6	[37.4,43.9]	21.8	[19.0,24.9]	11.8	[10.0,13.9]
San Marino	33	[25.7,41.2]	27.9	[22.3,34.3]	12.2	[8.5,17.3]	5.4	[3.4,8.4]	24.3	[18.2,31.5]	19.3	[13.7,26.6]	8.8	[5.6,13.6]	2.6	[0.9,7.3]
Slovakia					9.7	[7.7,12.2]	5.6	[4.2,7.5]					6.2	[4.7,8.0]	4.1	[3.0,5.6]
Slovenia	86.4	[83.0,89.2]	20.9	[16.7,25.9]			0		83.3	[80.0,86.1]	12.1	[9.9,14.8]			0	

Togo	16.4	[11.9,22.1]			1.4	[0.9,2.0]	0.5	[0.2,1.0]	15.9	[10.0,24.4]	23.7	[20.2, 27.2]	1	[0.5,1.9]	0.2	[0.1,0.5]
Congo	39.5	[34.7,44.4]	8.9	[7.3,10.7]	4.6	[3.7,5.6]	1.3	[0.9,1.9]	34.1	[29.6,38.9]	7.1	[5.2,9.5]	4	[2.8,5.6]	0.7	[0.4,1.2]
Ghana	14.2	[11.3,17.7]	8.2	[5.5,12.1]	5.5	[3.6,8.4]	0.8	[0.4,1.4]	13.9	[11.0,17.4]	8.7	[5.9,12.8]	6.1	[3.8,9.6]	1.6	[0.8,3.3]
Mauritania	36.4	[31.8,41.3]			17.5	[13.8,21.9]	5.3	[3.4,8.3]	29.9	[25.4,34.9]			19	[12.7,27.5]	7.6	[4.2,13.2]
Bolivia	52.2	[47.8,56.6]	16	[14.0,18.2]	10.8	[9.2,12.6]	3.9	[3.0,5.0]	44.9	[40.1,49.9]	9.2	[6.8,12.4]	5.7	[4.4,7.2]	2.2	[1.4,3.3]
El Salvador	37.3	[32.3,42.5]	10.3	[7.6, 13.1]	3.6	[2.8,4.6]	1.7	[1.2,2.4]	25.5	[22.1,29.2]	5.3	[3.7, 6.9]	2.5	[1.7,3.6]	1.4	[0.8,2.4]
Guatemala	42.6	[36.1,49.3]	11.7	[9.3,14.6]	6.5	[4.8,8.7]	3.8	[2.6,5.5]	36.9	[31.8,42.3]	11	[9.1,13.2]	5.3	[4.1,6.7]	2.3	[1.7,3.1]
Yemen	34.7	[27.8,42.3]	18.5	[16.2,21.0]	17.6	[15.7,19.7]	3.2	[2.1,4.7]	22	[16.2,29.3]	7.6	[4.7,11.9]	8.2	[6.0,11.1]	0.7	[0.3,1.6]
Morocco	48.8	[41.0,56.7]			6.9	[5.1,9.2]	1.2	[0.7,2.1]	40.2	[34.6,46.0]			3.8	[2.4,5.8]	0.3	[0.1,0.9]
Tunisia	60.7	[55.9,65.2]	9.6	[7.9,11.8]	7.5	[6.0,9.5]	3.1	[1.9,4.9]	48.5	[43.7,53.4]	3	[2.2,4.2]	2.1	[1.4,3.1]	0.1	[0.0,0.6]
Vanuatu	21.6	[17.9,25.7]	14.5	[11.4,18.2]	10.1	[7.4,13.6]	7.5	[5.2,10.7]	20.6	[17.4,24.3]	6.8	[4.8,9.4]	4.1	[2.7,6.0]	1.9	[1.1,3.1]
Cambodia	11.7	[8.8,15.5]	2.7	[2.0,3.6]	2.6	[2.0,3.4]	0.1	[0.0,0.3]	7.1	[5.6,9.1]	1.9	[1.2,2.9]	2.1	[1.5,3.0]	0.1	[0.0,0.3]
Kiribati	29.7	[26.5,33.1]	15.5	[12.2, 18.8]	15.7	[13.1,18.6]	7.6	[6.0,9.5]	22	[19.5,24.8]	8.5	[6.9, 10.2]	7.1	[5.2,9.7]	2.6	[1.5,4.5]
Lao Republic	18.1	[15.2,21.5]	89.5	[87.2,91.4]	5.9	[4.4,7.9]	2.3	[1.6,3.1]	15	[12.3,18.3]	93.5	[92.1,94.6]	3.6	[2.7,4.9]	0.4	[0.2,0.8]
Mongolia	56.7	[50.9,62.3]	14.6	[11.8,18.0]	5.7	[4.2,7.7]	1.8	[1.2,2.8]	48.2	[43.3,53.1]	5.1	[3.7,6.9]	1	[0.5,2.0]	0.2	[0.0,0.8]
Papua New Guinea	21.6	[18.0,25.6]			19.7	[16.1,23.8]	9.1	[6.8,11.9]	18	[15.5,20.8]			15.3	[12.1,19.1]	5.6	[3.6,8.7]
Philippines	49.7	[43.7,55.7]	16.8	[14.3,19.5]			0		37.2	[33.4,41.1]	7.6	[5.6,10.1]			0	
Ukraine	94.2	[91.4,96.1]	42.7	[38.5,47.1]	21.7	[18.1,25.9]	4.1	[2.7,6.1]	92.2	[90.0,94.0]	29.5	[25.8,33.4]	12.8	[10.4,15.7]	3.3	[2.3,4.7]
Georgia	81.5	[76.4,85.7]				[12.9,23.3]	6.1	[4.1,8.8]	76.3	[66.8,83.8]			6.4	[4.6,8.9]	0.6	[0.2,1.9]
Kosovo	42	[38.5,45.5]	10.6	[9.4,11.9]	6.2	[5.0,7.6]	1.6	[1.1,2.4]	31.6	[27.4,36.0]	4.2	[3.3,5.2]	2.7	[2.0,3.6]	0.5	[0.3,0.9]
Kyrgyzstan	47.9	[42.9,52.9]	7.7	[6.0, 9.4]	4.1	[3.0,5.5]	0.9	[0.6,1.5]	33.9	[29.1,39.1]	2.9	[1.9, 4.0]	1.6	[1.0,2.5]	0.1	[0.1,0.3]
Republic of Moldova	86.5	[83.6,89.0]	40.2	[35.7,45.0]	17.1	[14.9,19.6]	5.1	[3.8,6.7]	89	[86.8,90.9]	23.8	[21.5,26.1]	8.4	[6.8,10.4]	1.7	[1.2,2.5]
Nicaragua			16.7	[14.0, 19.3]	10	[8.6,11.5]	4.1	[3.3,5.0]			12.1	[9.6, 14.7]	7	[6.1,8.1]	2.1	[1.7,2.6]
Mauritius	69.1	[63.2,74.5]			18.3	[15.1,21.9]	10	[7.6,13.1]	39.9	[34.1,46.0]			4.6	[3.0,7.2]	1.6	[0.9,2.7]

Argentina	76.7	[69.0,83.0]	16.7	[11.3,23.9]	9.4	[6.0,14.4]	4.4	[2.8,6.9]	75.4	[65.7,83.1]	14.7	[10.4,20.2]	7	[4.8,9.9]	3.8	[2.4,5.8]
Belize	38.6	[32.9,44.7]	9.4	[6.9,12.7]	8.6	[6.6,11.2]	3.7	[2.4,5.5]	28.9	[24.1,34.2]	6.1	[4.5,8.4]	4	[3.0,5.5]	0.9	[0.5,1.7]
Cuba	52.6	[46.8,58.4]			7.1	[5.2,9.7]	3.3	[2.2,4.9]	46.6	[40.7,52.6]			4.6	[3.2,6.5]	1.7	[0.8,3.4]
Dominican Republic	72.7	[66.6,78.1]	26.2	[21.3,31.8]	12.7	[10.0,16.1]	2.2	[1.1,4.3]	62.2	[55.5,68.4]	14.6	[11.0,19.1]	8	[5.7,11.0]	0.8	[0.3,2.3]
Ecuador	54.7	[45.5,63.5]	20.9	[13.9,30.2]	12.9	[9.7,17.0]	4.2	[2.8,6.3]	46.2	[38.2,54.4]	13.9	[9.5,19.8]	8.6	[7.0,10.5]	3	[2.1,4.2]
Grenada	47.9	[41.2,54.6]			9.6	[7.4,12.3]	2.5	[1.6,3.9]	41.8	[36.7,47.1]			5.6	[4.0,7.6]	1.6	[1.0,2.5]
Guyana	26.3	[23.1,29.9]	9.6	[6.5,13.9]	8.8	[5.4,13.8]	3.1	[1.8,5.3]	18.8	[15.1,23.3]	5.5	[3.7,8.0]	6.7	[5.0,9.0]	1.5	[0.8,2.9]
Jamaica	46.2	[46.2,46.2]	19.3	[19.3,19.3]	15.2	[15.2,15.2]	5.3	[5.3,5.3]	42.2	[42.2,42.2]	13	[13.0,13.0]	9.7	[9.7,9.7]	2.4	[2.4,2.4]
Paraguay	78.3	[67.4,86.3]	26.0	[19.7, 32.3]	12.8	[10.3,15.7]	1.4	[1.0,2.1]	76.7	[64.9,85.5]	20.7	[16.6, 24.8]	9.9	[8.1,12.0]	1.8	[1.2,2.7]
Peru	54.7	[48.3,60.9]	14.6	[11.8, 17.5]	8.3	[6.7,10.2]	2.9	[2.0,4.1]	50.7	[44.7,56.6]	12.0	[9.8, 14.2]	5.3	[4.3,6.6]	1.6	[1.2,2.1]
Saint Lucia	52.5	[46.5,58.5]	19.6	[16.3,23.3]	14.4	[11.3,18.2]	3.7	[2.3,5.7]	42.3	[36.2,48.6]	11.6	[9.2,14.5]	6.6	[4.5,9.5]	2.1	[1.2,3.6]
Suriname	45.6	[40.1,51.1]			7.7	[5.7,10.4]	3.5	[2.5,4.9]	37.3	[32.0,42.9]			3.7	[2.7,5.2]	0.7	[0.4,1.4]
Iraq	58.6	[51.6,65.3]	20.3	[16.4,24.8]	12.8	[9.4,17.1]	6.5	[3.9,10.4]	47.5	[39.1,56.0]	9.8	[6.7,14.3]	4	[2.2,7.4]	1.6	[0.7,3.4]
Thailand	43	[35.4,50.9]	7.6	[5.4,10.7]	4.8	[3.6,6.5]	2.8	[2.1,3.6]	33.8	[28.1,40.1]	3	[1.9,4.7]	1.9	[1.0,3.5]	0.8	[0.3,2.1]
Indonesia	79.7	[77.2,82.1]	40.6	[36.7,44.6]	25.4	[22.7,28.2]	16.9	[15.0,19.1]	63.2	[59.2,67.0]	6	[5.0,7.0]	3.8	[3.2,4.6]	0.6	[0.4,0.9]
Maldives	64.2	[60.7,67.7]	26.3	[22.9,29.9]			0		53.7	[50.1,57.2]	11.2	[9.5,13.3]			0	
Fiji	39.4	[35.7,43.3]	17.7	[13.3,23.1]	16.3	[12.3,21.3]	4.9	[3.4,7.0]	29	[26.2,32.0]	7.2	[5.4,9.5]	9	[7.3,11.1]	1.5	[1.0,2.4]
Marshall Islands	30.5	[27.4,33.8]			24.9	[21.8,28.3]	13.1	[10.8,15.8]	26.1	[23.5,28.9]			13.1	[11.0,15.5]	3.9	[3.0,5.0]
Samoa	34.6	[29.8,39.8]	18.8	[15.6,22.5]	19.1	[16.0,22.8]	7.3	[5.6,9.4]	20.5	[16.9,24.7]	5.5	[4.1,7.5]	6.1	[4.5,8.1]	1.1	[0.5,2.5]
Albania	68.9	[64.0,73.4]			9.9	[8.4,11.7]	3.9	[2.7,5.6]	59.4	[55.2,63.5]			3.2	[2.4,4.3]	0.8	[0.4,1.5]
Belarus	80.5	[76.4,84.0]					0		83.3	[79.2,86.7]					0	
Bulgaria	87.4	[83.0,90.8]	22.5	[19.2,26.1]	12.6	[9.6,16.2]	5.9	[3.6,9.6]	89.7	[86.0,92.5]	20.6	[18.1,23.4]	8.9	[7.0,11.4]	5.6	[4.1,7.7]
Croatia	90.2	[87.5,92.4]	26.9	[20.7,34.3]	14.7	[11.2,19.1]	7.5	[4.8,11.4]	92	[89.1,94.2]	17.3	[13.8,21.5]	6.8	[5.1,9.1]	4.4	[3.0,6.5]
Kazakhstan	49.2	[40.0,58.5]	4.6	[2.6,7.9]	2.4	[1.3,4.2]	0.3	[0.1,0.8]	41.7	[30.1,54.3]	2	[1.1,3.6]	1.4	[0.8,2.4]	0.5	[0.2,1.3]

Macedonia	72	[68.7,75.1]			6	[4.7,7.5]	2.3	[1.6,3.3]	65.1	[61.5,68.5]			2.1	[1.6,2.8]	1	[0.6,1.5]
Montenegro	81.3	[78.5,83.9]					0		82	[79.0,84.6]					0	
Romania	22.7	[20.4,25.1]	16.8	[15.0,18.8]	9.6	[8.1,11.4]	3.5	[2.7,4.6]	16.5	[14.2,19.0]	11.5	[9.9,13.4]	5.5	[4.4,6.8]	2.3	[1.7,3.0]
Serbia			25.4	[23.5, 27.4]	8.4	[8.4,8.4]	4.1	[4.1,4.1]			21.1	[19.2, 22.9]	5.4	[5.4,5.4]	2.7	[2.7,2.7]
Srpska	83.2	[79.8,86.2]					0		90	[88.2,91.5]					0	
Saint Vincent					7.9	[5.9, 9.9]	1.4	[0.4, 2.3]					7.1	[4.5, 9.4]	2.4	[1.1, 3.7
Bosnia					17	[16.9, 19.1]	7.7	[6.2, 9.3]					6.5	[5.1, 7.9]	3.6	[2.4, 4.9]
